# Multiple myeloma with isolated central nervous system relapse after autologous stem cell transplantation: A case report and review of the literature

**DOI:** 10.3389/fonc.2022.1027585

**Published:** 2022-11-25

**Authors:** Xian Li, Weiqin Wang, Xiaohong Zhang, Yun Liang

**Affiliations:** Department of Hematology, The Second Affiliated Hospital, College of Medicine, Zhejiang University, Hangzhou, China

**Keywords:** multiple myeloma, isolated central nervous system relapse, autologous stem cell transplantation, pathogenesis and treatment, case report

## Abstract

Patients with multiple myeloma (MM) rarely present with central nervous system (CNS) involvement as a manifestation of extramedullary disease (EMD), a condition that is associated with poor prognosis. CNS relapse without evidence of systemic involvement is even rarer, and there is no standardized treatment because there are only few case reports. We present a 47-year-old female who was diagnosed with nonsecretory multiple myeloma (NSMM) 9 years previously. She had a complete remission after receiving aggressive therapies, including high-dose chemotherapy and autologous stem cell transplantation (ASCT). However, after 7 years of progression-free survival, she had CNS relapse without evidence of systemic involvement. We switched to a salvage regimen consisting of high-dose methotrexate with lenalidomide. She achieved rapid clinical improvement, with a reduction in cerebrospinal fluid plasmacytosis of more than 80%, and no notable side effects. Our description of this unique case of a patient with MM and isolated CNS relapse after ASCT provides a reference for physicians to provide more appropriate management of these patients. We also reviewed previously reported cases and summarized the outcomes of isolated CNS relapse after ASCT, and discuss the pathogenesis and possible treatment strategies for MM with isolated CNS relapse.

## Introduction

Multiple myeloma (MM) is characterized by the monoclonal proliferation of plasma cells (PCs) in bone marrow (1). Despite the use of established treatments followed by autologous stem cell transplantation (ASCT) and improvements in patient outcomes during recent years, MM is still incurable (2). Relapse in most patients is characterized as a medullary monoclonal proliferation, and 3.4% to 35% of these patients present with extramedullary disease (EMD) (3). Central nervous system (CNS) involvement is a very rare aggressive presentation of EMD, and occurs in only about 1% of patients (4). CNS relapse without evidence of systemic involvement is even rarer, with only few case reports, and these patients face a very poor prognosis, with a median survival time less than 6 months (5).

The present study describes a female who had MM with isolated CNS relapse after ASCT, and faced a poor prognosis despite the use of aggressive therapy. There is no standard treatment for CNS localization of multiple myeloma (CNS-MM) (4, 6) due to the rarity of this presentation. Thus, we also conducted a literature review to summarize the outcomes of other MM patients who had isolated CNS relapse after ASCT and examined the pathogenesis and possible treatment strategies for this condition.

## Case report

A 38-year-old female with lumbago was diagnosed with nonsecretory multiple myeloma (NSMM) in December 2012. At that time, bone marrow specimens indicated 74% infiltration of plasma cells, and flow cytometry analysis showed abnormal plasma cells, which were positive for CD38, CD56, CD138, and cytoplasmic λ light-chain. Serum immunofixation (IFE) showed no detectable monoclonal component, a blood examination showed no anemia or renal dysfunction, and the levels of lactate dehydrogenase (LDH) and β_2_ microglobulin (β_2_-MG) were normal. Whole body bone imaging showed diffuse abnormal signals in the ribs, spinal vertebrae, and ilium. These findings led to a diagnosis of NSMM, with stage I based on the International Staging System (ISS) and stage IIIA based on the Durie-Salmon (DS) staging system. The patient received 4 courses of bortezomib, dexamethasone, and thalidomide (VDT) and achieved a complete response (CR).

After a treatment-free period of 4 months, she presented again with low backache. Bone marrow flow cytometry indicated that 6.5% of the plasma cells were abnormal, indicative of medullary recurrence. She then received 8 courses of different chemotherapies: 4 courses of vincristine, doxorubicin, and dexamethasone (VAD); 3 courses of vincristine, dexamethasone, cyclophosphamide, and thalidomide (VDCT); and 1 course of thalidomide, dexamethasone, cis-platin, doxorubicin, cyclophosphamide, and etoposide (DTPACE). After treatment, she achieved a partial response (PR) with regression of bone pain and 1% plasma cells in bone marrow.

In July 2014, she was given ASCT with preconditioning using semustine, busulphan, and etoposide (Me-CCNu + Bu + VP-16) and maintained a PR. However, 5 months after ASCT, she developed right-lower limb pain. Whole body bone imaging at that time showed a new focus in the right femoral region, and the bone marrow had 14% plasma cells with a normal level of the M protein based on immunofixation electrophoresis (IFE). Thus, melphalan and prednisolone (MP) therapy was initiated. There were no detectable myeloma cells in the bone marrow after 6 courses of this therapy. Thalidomide (100 mg orally) maintenance therapy was then administered for 2 years, and she had no further relapse.

**Figure 1:**
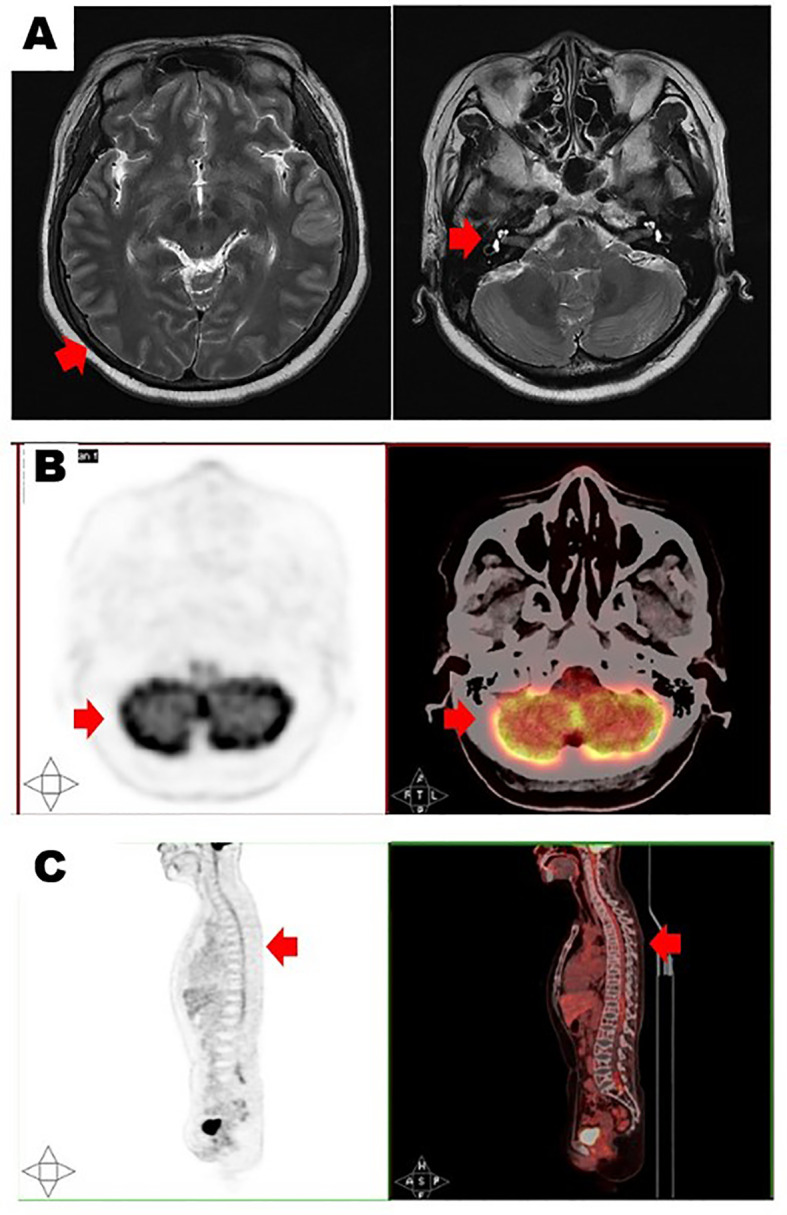
Brain magnetic resonance imaging **(A)** showed cerebrospinal meninges (left, red arrow) and auditory nerve thickening (right, red arrow). Positron emission tomography/computerized tomography in transverse section **(B)** and longitudinal section **(C)** showed multiple cerebrospinal meninges with increased 18F-flurodeoxyglucose metabolism (red arrows).

**Figure 2:**
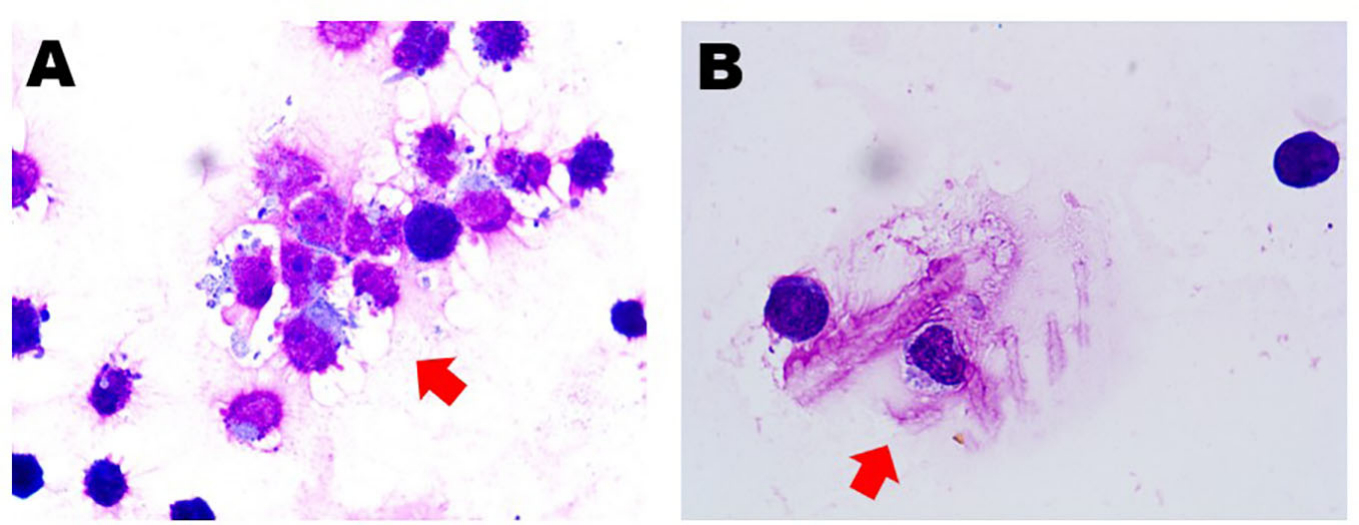
Cerebrospinal fluid smear showed the presence of abnormal plasma cells (red arrows) before **(A)** and after **(B)** salvage therapy.

In May 2022, she presented again and reported the sudden onset of dizziness, staggering gait, and loss of hearing. Physical examination revealed that she had clear poor hearing. The muscular strength tension of limbs was normal. Physiological reflexes were existent without any pathological ones. No enlargement of lymph nodes, liver, or spleen was found. Brain magnetic resonance imaging (MRI) showed cerebrospinal meninges and auditory nerve thickening (**Figure 1A**). Positron emission tomography/computerized tomography (PET/CT) showed multiple cerebrospinal meninges with increased ^18^F-flurodeoxyglucose metabolism, but no other site of disease involvement (**Figures 1B, C**). Further examination showed she had no abnormalities in the hemogram, M-protein level, renal function, LDH level, and β_2_-MG level. A bone marrow analysis showed no chromosomal abnormalities and no increased number of abnormal plasma cells. However, her cerebrospinal fluid (CSF) was positive for plasma cells (**Figure 2A**), and a lumbar puncture showed the CSF had a protein content of 213.8 mg/dL (normal range: 20–40), glucose of 50 mg/dL (normal range: 50–60), and 42×10^6^ nucleated cells/L (normal range: 0–8×10^6^). These findings indicated that the relapse was localized to the CNS.

**Figure 3:**
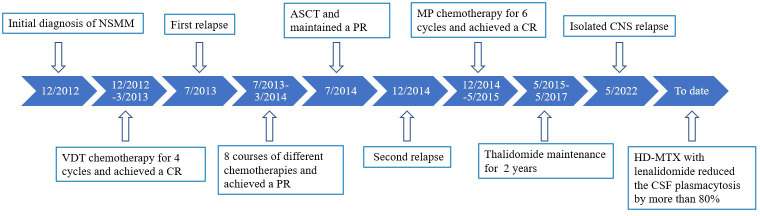
Timeline of the patient’s diagnosis and treatments.

We advised high-dose methotrexate (HD-MTX) therapy with lenalidomide (25 mg orally). After one course of salvage therapy, she achieved rapid clinical improvement without any notable side effects, such as hematological toxicity or peripheral neuropathy. Furthermore, this treatment reduced the CSF plasmacytosis by more than 80% (**Figure 2B**). The timeline of the patient is summarized in **Figure 3**. 

## Discussion

ASCT after induction therapy is a common standard treatment for eligible MM patients because it can induce durable remission and improve long-term survival. Nonetheless, MM is still an incurable disease. Although most patients who experience relapse have proliferation of monoclonal plasma cells, mainly in the bone marrow, about 3.4% to 35% of these patients present with EMD (1, 3). CNS involvement is a specific presentation of extramedullary extraosseous, and occurs in only about 1% of patients (4). The median survival time of these patients is only 4 to 7 months, even when aggressive therapy is given (4, 7). CNS relapse without evidence of systemic involvement after ASCT is even rarer in patients who have MM, and there are only a few case reports in the literature.

Certain clinical factors are associated with increased risk of CNS-MM, including lambda subtype, elevated LDH, elevated β_2_-MG, EMD, plasma cell leukemia, and chromosomal abnormalities (deletion of 17p or 13q) (4, 8, 9). We performed a comprehensive search of the literature and identified 14 cases (**Tables 1**, **2**). Most of these patients had ISS stage III disease at diagnosis, but the myeloma subtype was variable. There were more patients with high LDH and β_2_-MG levels than with normal levels. Only one patient had plasma cell leukemia. The median time from ASCT to CNS disease was 6 months (range: 2.5–84), and most patients died after developing CNS disease, with a median survival post-CNS relapse of 6 months (range: 0.3–29). Cytogenetic results were available in 7 patients: 4 patients had 17p deletion (17p-), 2 patients had 1q21 amplification (1q21+), and 2 patients had translocation (4, 14). These cytogenetic abnormalities may be related to isolated CNS relapse after ASCT for MM. This is consistent with the observations from previous studies (4). One cohort study showed that deletion of chromosome 17p13,1 (p53) was present in 89% of the CNS-MM patients and associated with metastatic features of myeloma cells (20). Moreover, investigators found that amplification of 1q21 was associated with disease progression and poor prognosis in MM despite the use of novel regimens (21). Patients with 1q21+ showed a high incidence of aggressive features, including an unusually high CNS involvement incidence (11%) and early onset of CNS disease (22). Our patient, who had bone marrow expression of CD56 had no EMD or circulating plasma cells at baseline. Our patient differed from other previously described patients in that she had normal levels of LDH and β_2_-MG and no cytogenetic abnormalities. Because factors that apparently increase the risk for CNS involvement were not present in our patient, we examined the possible reasons why she developed such aggressive disease.

**Table 1:** Published case reports of patients with multiple myeloma who had isolated CNS relapse after ASCT.

Patient No. (Reference)		1 (10)	2 (11)	3 (12)	4 (13)	5 (14)	6 (15)	7 (15)	8 (16)
**Age, years**		39	55	32	58	29	49	66	66
**Gender**		Male	Male	Female	Male	Male	Male	Male	Male
**Myeloma type**		IgA-λ	IgG-κ	IgA-κ	IgA-κ	IgG-κ	IgG-λ	IgA-λ	IgG-κ
**Stage**									
	DS	IIIB	IIIB	IIIA	IIIB	IIIB	IIIA	IIIA	IIIB
	ISS					III	III	III	III
**Plasma cell CD56 status**		NA	NA	NA	NA	+	+	–	–
**LDH**		>ULN	>ULN	>ULN	NA	NA	NA	NA	NA
**β_2_-MG**		≤ULN	NA	>ULN	>ULN	>ULN	NA	NA	NA
**Cytogenetic abnormalities**		NA	NA	NA	NA	NA	17p-, 1q21+	1q21+	NA
**Plasma cell leukemia**		yes	no	no	no	no	no	no	no
**Treatments prior to ASCT, n**		5	3	4	6	4	4	4	4
**High-dose therapy**		Mel	Mel	Mel	Bu/Mel/CY	Mel	Mel	Mel	NA
**Time to relapse post-ASCT**		3 months	3 months	10 weeks	7 years	6 months	9 months	6 months	8 months
**Parenchymal**		yes	no	no	yes	yes	yes	yes	NA
**Treatment for CNS-MM**		IT	BCNU/CY/IT/RT/ASCT	IT	IT/Dexa	CTAD/IT/RT	Surgery/RT DPACE/RD/DVD	IT/RT	IT/RT/Dexa
**Best response to CNS-MM treatment**		PD	CR	PD	CR	SD	PR	CR	CR
**Survival post-CNS relapse**		9 days	7 months	8 days	11 months	3 months	29 months	12 months	10 months
**Patient No. (Reference)**		**9 (** 16)	**10 (** 17)	**11 (** 8)	**12 (** 18)	**13 (** 19)	**14 (current case)**		
**Age, years**		40	58	56	62	46	38		
**Gender**		Female	Male	Female	Female	Female	Female		
**Myeloma type**		IgA-κ	IgG-κ	IgA-κ	IgG-λ	IgA-λ	nonsecretory		
**Stage**									
	DS	IIIB	IIIB		IIIB	IIIB	IIIA		
	ISS	III	III	III			I		
**Plasma cell CD56 status**		+	NA	NA	+	+	+		
**LDH**		NA	>ULN	NA	≤ULN	NA	≤ULN		
**β_2_-MG**		NA	>ULN	NA	≤ULN	>ULN	≤ULN		
**Cytogenetic abnormalities**		17p-, t (4;14)	17p-, t (4;14)	hyperdiploid karyotype	17p-	NA	None		
**Plasma cell leukemia**		no	no	no	no	no	no		
**Treatments prior ASCT, n**		3	4	3	4	2	12		
**High-dose therapy**		NA	Mel	NA		Mel	Bu		
**Time to relapse post-ASCT**		8 months	5 months	4 months	7 years	6 months	7 years		
**Parenchymal**		NA	no	yes	yes	yes	no		
**Treatment for CNS-MM**		Dexa	DKBP-BD	VTD-PACE	IT/Dexa/PD	Chemotherapy*/IT/RT	HD-MTX/lenalidomide		
**Best response to CNS-MM treatment**		SD	CR	PR	CR	PR			
**Survival post CNS relapse**		2 months		2 months	11 months	5 months			

DS, Durie Salmon Staging system; ISS, International Staging System; LDH, lactate dehydrogenase; β_2_-MG, β_2_ microglobulin; ASCT, autologous stem cell transplatation; CNS-MM, central nervous system localization of multiple myeloma; NA, not available; ULN, upper limit of normal; PD, progressive disease; CR, complete response; SD, stable disease; PR, partial response; Mel, melphalan; CY, cyclophosphamide; BU, busulfan; IT, intrathecal chemotherapy; BCNU, carmustine; RT, radiotherapy; MP, melphalan and prednisolone; HDT, high dose therapy; Dexa, dexamethasone; CTAD, cyclophosphamide, thalidomide, adriamycin, and dexamethasone; DPACE, dexamethasone, cisplatin, doxorubicin, cyclophosphamide and etoposide; RD, lenalidomide and dexamethasone; DVD, daratumumab, bortezomib, and dexamethasone; DKBP,BD, dexamethasone, carfilzomib, bendamustine, pomalidomide, clarithromycin, and daratumumab; VTD-PACE, bortezomib, thalidomide, dexamethasone, cis,platin, doxorubicin, cyclophosphamide, and etoposide; PD, pomalidomide and dexamethasone; Chemotherapy*, topotecan, temozolomide and dexamethasone; HD-MTX, high-dose methotrexate.

**Table 2:** Summary of multiple myeloma cases who had isolated CNS relapse after ASCT (n=14).

Characteristic	n	%
**Gender**		
Male	9	64
Female	5	36
**Age, median years (range)**	52 (29,66)	
**Myeloma type**		
IgA-λ	3	21
IgA-κ	4	29
IgG-λ	2	14
IgG-κ	4	29
Nonsecretory	1	7
**Cytogenetics**		
17p-	4	29
1q21+	2	14
t (4;14)	2	14
Not evaluated	7	
**LDH**		
>ULN	4	29
≤ULN	2	14
Not evaluated	8	
**β_2_-MG**		
>ULN	5	36
≤ULN	3	21
Not evaluated	6	
**Plasma cell leukemia**	1	7
**Time to relapse post ASCT, median months (range)**	6 (2.5,84)	
**Treatment for CNS-MM**		
Intrathecal	9	64
Radiotherapy	6	43
Proteasome inhibitors	3	21
Immunomodulatory drugs	6	43
Anti-CD38 monoclonal antibody	2	14
ASCT	1	7
**Survival post CNS relapse, median months (range)**	6 (0.3,29)	

ULN, upper limit of normal.

The mechanism leading to isolated CNS relapse post-ASCT is uncertain. One hypothesis is that malignant plasma cells are transmitted by blood or plasma cell precursors, and then spread in the cerebrospinal meninges. In the past decade, therapies using novel agents and ASCT have improved the progression-free survival of MM patients, and it seems likely that this has led to the appearance of new patterns of relapse. The downregulation of CD56 adhesion molecules after first-line therapy could allow MM cells to escape the bone marrow environment and establish distant plasma cell metastasis, including in the CNS (18). Patients with plasma cell leukemia have abnormal plasma cells in the circulating blood, and the presence of these circulating plasma cells increases the risk of hematogenous spread. This supports our first hypothesis that malignant plasma cells are transmitted in the blood, and then spread to the cerebrospinal meninges (23). A second hypothesis is that plasmacytoma infiltrated adjacent skull lytic lesions. These patients mainly have parenchymal infiltration, varying from 39% to 65% in some cohorts (5, 24). Finally, a series of reports showed that clonal heterogeneity could play a role in CNS-MM. In particular, high dose chemotherapy for ASCT might select for extramedullary drug-resistant clonal populations, thus leading to relapse without bone marrow involvement (14, 25, 26). Our patient received first-line ASCT after aggressive therapy, and had none of the factors associated with risk for CNS involvement at baseline. After our patient achieved a 7-year progression-free survival, the selection of plasma cells with an atypical homing behavior and the absence of immunoglobulin secretion may have led to the isolated CNS relapse. We hypothesize that her relapse may have been from a new clone, rather than the clone responsible for the initial diagnosis.

There is currently no standard treatment for CNS-MM. Traditional therapeutic strategies include chemotherapy, surgery, radiotherapy, and intrathecal injection, but evidence supporting their efficacy is limited and durable remission is rare (27). Previous studies of systemic chemotherapy agents (methotrexate, cytarabine, edabixin, azathioprine and thiotepa) that can penetrate the blood-brain barrier (BBB) may provide a rapid therapeutic effect (8, 28). However, due to their CNS toxicity and low efficacy in MM patients who have chromosome 17p-, treatments consisting of traditional chemotherapy drugs alone are insufficient. Given the known radiosensitivity of malignant plasma cells, craniospinal irradiation is frequently used to treat parenchymal CNS-MM lesions (29). Although this treatment modality is associated with a statistically significantly longer survival (9), hematologic toxicity is a potential concern, especially in the cases who prior exposure to several myelosuppressive chemotherapy agents and ASCT (30).

Although novel agents have improved the outcomes of patients with CNS-MM (31), most conventional anti-myeloma drugs have relatively poor CNS penetration. A literature review of the penetration of novel myeloma-active drugs into the CSF reported that some immunomodulatory drugs (IMiDs) entered the CSF. For example, thalidomide can be detected in CSF after oral administration (32) and the lenalidomide and pomalidomide concentrations in CSF can reach 11% to 49% of the peak concentration in blood. Thus, these drugs may have good CSF activity against lymphoma and myeloma when there is CNS involvement (33–36). In addition, similar studies showed that one-third of lenalidomide-resistant patients still responded to pomadodomide, particularly those with MM with chromosome 17p- and/or translocation (4, 14) (37, 38).

Few proteasome inhibitors can penetrate the BBB, limiting their efficacy in patients with CNS-MM (27). Marizomib and carfilzomib are novel next-generation proteasome inhibitors that can pass through the BBB and may be effective in CNS-MM. For example, an animal study of radiolabeled marizomib reported the CNS level was 30% of that in the blood (39). Case reports (40) showed that marizomib provided clinical and radiological improvements, so it may be an effective approach for treatment of CNS-MM. Some case series also reported that carfilzomib was effective in the clearance of myeloma cells from CSF (41).

Some studies examined the ability of monoclonal antibodies to improve the outcomes of patients with CNS-MM. Although the penetration of systemic daratumumab (anti-CD38 monoclonal antibody) into the CNS was limited, it produced durable responses in some case reports. It is possible that the BBB becomes more permeable in certain disease states, such as when there is disruption of the meninges (28, 42).

In addition to monoclonal antibodies, the recently developed B-cell maturation antigen, chimeric antigen receptor T cell (BCMA CAR-T) therapy is a novel treatment strategy for relapsed/refractory(R/R) CNS-involved MM. For example, Wang et al. identified the presence of BCMA CAR-T cells in CSF (43). The mechanisms responsible for the higher CD4/CD8 ratio in CSF than in peripheral blood may regulate the penetration of CD4 + and CD8 + CAR-T cells across the BBB and their proliferation in CSF to kill myeloma cells. Several studies investigated the effects of BCMA CAR-T cells on CNS-MM patients and reported remarkable clinical remissions (43, 44). Closer monitoring of patients may help in the early identification of CAR-T neurotoxicity, thus making immune effector cell-associated neurotoxicity syndrome (ICANS) more predictable and controllable (45). BCMA CAR-T therapy appears to be a safe and effective for treatment for R/R CNS-MM, but the duration of remission is a remaining problem.

Although the optimal therapy for CNS-MM is uncertain because of the rarity of this condition, aggressive management is necessary. Examination of individualized combinations of chemotherapy, targeted drugs, monoclonal antibodies, CAR-T cells, and local therapy could lead to further improvements of outcomes.

## Conclusion

Our study describes a case of CNS-MM following ASCT, with no evidence of systemic involvement. High dose methotrexate and lenalidomide (which can cross the BBB) produced a rapid response and effectively cleared myeloma cells from the CSF, but the duration of this remission must be addressed. Isolated CNS relapse after ASCT in MM is extremely rare. Even with novel therapies, the survival time after CNS-MM remains poor, and the optimal method for management of these patients is an open question because of the rarity of this condition. Further studies are required to identify factors associated with CNS relapse after ASCT and the underlying mechanism, and to determine improved methods of prophylaxis and management.

## Data availability statement

The original contributions presented in the study are included in the article/supplementary material. Further inquiries can be directed to the corresponding author.

## Ethics statement

The studies involving human participants were reviewed and approved by The Medical Ethics Committee of The Second Affiliated Hospital, College of Medicine, Zhejiang University. The patients/participants provided their written informed consent to participate in this study. Written informed consent was obtained from the individual(s) for the publication of any potentially identifiable images or data included in this article.

## Author contributions

XL, WW, and YL contributed to the design and conception of the study; XL and WW contributed to data collection; XL contributed to writing the initial drafting of the manuscript; XZ and YL reviewed and edited the original draft. All authors contributed to manuscript revision and read and approved the submitted version.

## Conflict of interest

The authors declare that the research was conducted in the absence of any commercial or financial relationships that could be construed as a potential conflict of interest.

## Publisher’s note

All claims expressed in this article are solely those of the authors and do not necessarily represent those of their affiliated organizations, or those of the publisher, the editors and the reviewers. Any product that may be evaluated in this article, or claim that may be made by its manufacturer, is not guaranteed or endorsed by the publisher.
